# A cluster-randomized trial of workplace ergonomics and neck-specific exercise versus ergonomics and health promotion for office workers to manage neck pain – a secondary outcome analysis

**DOI:** 10.1186/s12891-021-03945-y

**Published:** 2021-01-12

**Authors:** Venerina Johnston, Xiaoqi Chen, Alyssa Welch, Gisela Sjøgaard, Tracy A. Comans, Megan McStea, Leon Straker, Markus Melloh, Michelle Pereira, Shaun O’Leary

**Affiliations:** 1 The University of Queensland, School of Health and Rehabilitation Sciences, St Lucia, Brisbane, Queensland 4067 Australia; 2 The University of Queensland, Centre for Health Services Research, Brisbane, Australia; 3 University of Southern Denmark, Department of Sport Science and Clinical Biomechanics, Faculty of Health Sciences, Odense, Denmark; 4 Curtin University, School of Physiotherapy and Exercise Science, Perth, Australia; 5 Zurich University of Applied Sciences, School of Health Professions, Institute of Health Sciences, Winterthur, Switzerland; 6 Curtin University, Curtin Medical School, Perth, Australia; 7 The University of Western Australia, UWA Medical School, Perth, Australia; 8 National Healthcare Group, Health Services and Outcomes Research, 3 Fusionopolis Link #03-08, Singapore; 9 Royal Brisbane and Women’s Hospital, Department of Physiotherapy, Metro North Hospital Health Service, Brisbane, Australia

**Keywords:** Neck pain, Exercise, Health promotion, Workplace, Ergonomics

## Abstract

**Background:**

Neck pain is prevalent among office workers. This study evaluated the impact of an ergonomic and exercise training (EET) intervention and an ergonomic and health promotion (EHP) intervention on neck pain intensity among the All Workers and a subgroup of Neck Pain cases at baseline.

**Methods:**

A 12-month cluster-randomized trial was conducted in 14 public and private organisations. Office workers aged ≥18 years working ≥30 h per week (*n* = 740) received an individualised workstation ergonomic intervention, followed by 1:1 allocation to the EET group (neck-specific exercise training), or the EHP group (health promotion) for 12 weeks. Neck pain intensity (scale: 0–9) was recorded at baseline, 12 weeks, and 12 months. Participants with data at these three time points were included for analysis (*n* = 367). Intervention group differences were analysed using generalized estimating equation models on an intention-to-treat basis and adjusted for potential confounders. Subgroup analysis was performed on neck cases reporting pain ≥3 at baseline (*n* = 96).

**Results:**

The EET group demonstrated significantly greater reductions in neck pain intensity at 12 weeks compared to the EHP group for All Workers (EET: β = − 0.53 points 95% CI: − 0.84– − 0.22 [36%] and EHP: β = − 0.17 points 95% CI: − 0.47–0.13 [10.5%], *p*-value = 0.02) and the Neck Cases (EET: β = − 2.32 points 95% CI: − 3.09– − 1.56 [53%] and EHP: β = − 1.75 points 95% CI: − 2.35– − 1.16 [36%], *p* = 0.04). Reductions in pain intensity were not maintained at 12 months with no between-group differences observed in All Workers (EET: β = − 0.18, 95% CI: − 0.53–0.16 and EHP: β = − 0.14 points 95% CI: − 0.49–0.21, *p* = 0.53) or Neck Cases, although in both groups an overall reduction was found (EET: β = − 1.61 points 95% CI: − 2.36– − 0.89 and EHP: β = − 1.9 points 95% CI: − 2.59– − 1.20, *p* = 0.26).

**Conclusion:**

EET was more effective than EHP in reducing neck pain intensity in All Workers and Neck Cases immediately following the intervention period (12 weeks) but not at 12 months, with changes at 12 weeks reaching clinically meaningful thresholds for the Neck Cases. Findings suggest the need for continuation of exercise to maintain benefits in the longer term.

**Clinical trial registration:**

hACTRN12612001154897 Date of Registration: 31/10/2012.

**Supplementary Information:**

The online version contains supplementary material available at 10.1186/s12891-021-03945-y.

## Background

Office workers have among the highest prevalence of neck symptoms (18–63% per year) compared to other occupations [1] and other bodily locations [2]. Due to the amount of time an individual spends at the workplace and because of the strong link between health and productivity, the workplace is becoming the arena for many health initiatives. The most common workplace-based interventions tested to address neck problems in office workers tend to include either exercise or ergonomic changes. A recent review of workplace-based interventions found moderate quality evidence that strengthening exercises were effective in reducing neck pain in office workers who were symptomatic, but did not demonstrate effectiveness in a population of office workers that included those with and without symptoms [3]. Similarly, van Eerd et al. [4] found strong evidence for resistance training programs recommending these be implemented to help prevent and manage work-related upper extremity musculoskeletal symptoms and disorders.

Ergonomic interventions are endorsed by local health and safety regulatory authorities [5, 6], but the scientific evidence of these interventions for the prevention and management of neck symptoms is mixed [3, 7, 8]. Chen et al. [3] found limited evidence that multiple workstation adjustments may be effective in office workers who are symptomatic with conflicting and low quality evidence for a general population of office workers. In contrast, van Eerd et al. [4] found moderate evidence of a positive effect for forearm supports and mixed evidence that ergonomic training plus workstation adjustments were effective. The discrepancies could be due to the difference in outcomes (neck vs upper extremity pain), and occupational groups included (office workers vs all occupations). Nevertheless, it is possible that a combination of the most common interventions, exercise and ergonomic, will yield benefits for the office worker with neck pain than either alone. This was the case in a recent six month randomized trial which found the combination of exercise and ergonomic modifications and exercise alone were effective in reducing the severity of neck pain in office workers symptomatic at baseline compared with a control group [9]. Indeed, the recommendation from that study was that therapists should include exercise training to achieve long-term benefits rather than rely solely on ergonomic modifications.

There has recently been a move in comparative effectiveness research to identify the most efficient and effective public health interventions [10], providing better evidence to guide decision makers (in this case employers) in improving health outcomes for all employees. Comparing interventions in the general office worker population (including symptomatic and asymptomatic office workers) is informative to employers to determine if they are equitable and financially efficient from preventative and symptom management perspectives. There is current evidence supporting workplace-based strengthening exercise interventions in symptomatic office workers [3, 4] with emerging evidence for these interventions among the general office worker population [11]. Furthermore, there is a limited number of trials evaluating the effects of a combined strengthening exercise and ergonomic intervention [3]. This study aimed to merge the interventions with the strongest evidence amongst office workers for addressing neck pain (exercise) [3], with current health and safety regulations (ergonomic) [6].

The aim of this study was to evaluate the immediate (after 12 weeks intervention) and long-term (12-month follow-up) effects of a combined exercise and ergonomic intervention on neck pain in a general office worker population, and among office workers symptomatic at baseline. The novelty of this approach is the combination of common interventions delivered to all workers with the focus of this paper on the secondary prevention of neck pain. To be consistent with the trend for comparative effectiveness research to inform public health interventions [10], a general health promotion intervention was combined with the ergonomic intervention as the comparator. This comparator intervention could serve as a more scalable alternative, requiring less specialised services to deliver than neck-specific exercises, and if found effective in preventing and managing neck pain, could benefit all employees (not only those with neck pain). We hypothesized that the combined ergonomics and exercise training (EET) would be more effective than the combined ergonomics and health promotion (EHP) in reducing neck pain intensity in a general office worker population, as well as in those with neck pain.

## Methods

### Design and sample size

A prospective, two-arm 12-month cluster-randomized trial (ACTRN12612001154897) was conducted from May 2013 to July 2016. The protocol has been published [12], and follows CONSORT guidelines [13]. With the primary outcome (productivity loss) reported previously [14], this report focuses on one secondary outcome: neck pain intensity. Written, informed consent was obtained from all participants, and ethical approval granted by The University of Queensland Human Research Ethics Committee (#2012001318). Procedures were in accordance with ethical standards of the institutional committee and the Helsinki declaration and its later amendments [15].

Individuals and organisations were not reimbursed for their participation. The target sample size (*n* = 640) was based on the primary health-related productivity outcome, and 10% attrition [12]. During recruitment, higher attrition of approximately 25% was observed, due to more than expected organisational restructuring, and the target sample size was revised to 720 [14]. To enhance retention in the study, health-enhancing incentives were offered to some participants for high adherence during the 12 week intervention period.

### Participants

Employees were recruited from 14 public (*n* = 9) and private (*n* = 5) metropolitan-located organisations in Brisbane, Australia. Organisations were from public service administration (7), manufacturing/construction (4), higher education (1), insurance (1) and local government (1). Internal email invitations (and three follow-up emails) were sent from the on-site liaison of the participating organisation for recruitment, and on-site information sessions by the research team were conducted. Eligible participants were office workers aged ≥18 years, working ≥30 h/week. Exclusion criteria were any specific medical conditions (e.g. congenital cervical abnormalities, stenosis, radiculopathy) and contraindications to exercise (e.g. uncontrolled hypertension, angina), and anticipated prolonged absence from work during the study period [12].

### Randomisation

Eligible participants were clustered by building, floor, or work unit in descending order. The optimal cluster size was pre-determined (5–8) to ensure adequate supervision and facilitation for the EET. In open planned offices, larger or smaller clusters were formed to reduce the risk of contamination. Clustering ensures homogeneity within- and heterogeneity between-clusters. An even number of clusters were created within each organisation. A statistician blinded to the identity of the individuals assigned the clusters by simple random allocation using computer-generated block randomisation (in blocks of four) to either the EET or the EHP group. All participants within the same cluster were randomized to the same intervention and notified of their assignment by the project manager after completion of baseline assessments but before interventions commenced. This was repeated at each organisation.

### Interventions

Detailed trial methods have been published [12]. Intervention sessions and assessments occurred during work time with reminders set up in online calendars. All participants received an individualised 30–45-min workstation assessment by a blinded health professional prior to group allocation. An observational checklist (37 items) of the physical workstation across five domains (Chair - 7 items; Desk - 9 items; Keyboard and Mouse – 8 items; Screen - 5 items; Telephone - 3 items; Environment – 5 items) was used with moderate-to-good inter-rater reliability demonstrated [16]. Post-assessment, individual adjustments to the chair (height and backrest), monitor (e.g. reposition of monitor/s) and other workstation items were provided as needed. Some participants required specialised equipment (e.g. new chair, footrest or headset) which were sourced onsite or funded through the study. Advice or education (e.g. take frequent breaks) were also provided as required. .

#### Ergonomics and neck-specific exercise training

EET participants additionally received a progressive neck-specific, group-based exercise program for 12 weeks, consisting of three 20-min sessions per week [12]. The exercises included postural facilitation, upper neck flexion exercise (warm up for each session), and five main progressive resisted exercises performed in cycles of three exercises/session (i.e. neck flexion, neck extension, reverse flies, front and side arm raise to 90^0^). Training load for each individual was based on their one-repetition maximum (1-RM) recorded prior to commencement for the shoulder and neck exercises. This load was regularly re-evaluated by the intervention physiotherapist to ensure appropriate exercise progression. Participants were supervised by the physiotherapist for two sessions in the first week and, subsequently, one session each week to enhance intervention adherence and ensure efficient, correct, and safe execution of exercises. Paper diaries were issued to all participants with the exercises described in detail. These diaries served to remind participants of the exercises to be performed at each session and to record exercise intensity. On completion, participants were asked to continue the exercise training (explained in the diary) and were provided with resistance bands, a proposed fortnightly training schedule and instructions on how to perform exercises at home.

#### Ergonomics and health promotion

EHP participants additionally received one weekly 60-min health promotion session for 12 weeks, facilitated by a health professional. Sessions were hour-long in duration to ensure parity of intervention contact across groups. Topics discussed related to healthy eating, alcohol and tobacco use, stress and conflict management, mental health and lifestyle using resources provided by a government-based health department. General fitness topics were mentioned but specific information regarding neck exercises was not included.

### Outcome measures

All participant-reported outcomes were collected by an online survey. Neck pain intensity was evaluated at baseline, immediately post-intervention (12 weeks), and at 12-month follow-up using a body diagram and scale from 0 (no pain) to nine (worst possible pain) during the last seven days with the question, ‘*Please rate the degree of complaints (symptoms, pain, discomfort, ache) experienced in the body regions indicated above (picture of body regions)”* [17]. Neck cases (i.e. those symptomatic at baseline) were defined as those who scored ≥3 [18]. Reductions of ≥30% neck pain intensity from baseline were considered moderately clinically important amongst neck cases [19]. This self-report scale has been shown to be valid [18] and reliable [20] among workers with clinical signs of neck-shoulder disorders.

### Potential confounding variables recorded at baseline

#### Demographic, general and global health measures

These included age, gender, body mass index, number of comorbidities, highest level of education, receipt of workers’ compensation or healthcare professional consultation for neck/shoulder symptoms in the previous 12 months, and/or took medication for neck/shoulder symptoms in the previous four weeks. Psychological distress was assessed with the Kessler 6 scale (K6 short-form), with higher scores indicating higher psychological distress (0–24 minimum to maximum) [21, 22]. Health-related quality of life was assessed with the Assessment of Quality of Life (AQOL) 6D, a valid and reliable measure consisting of 20 questions (six dimensions: independent living, relationships, mental health, coping, pain and senses), with higher scores indicating higher quality of life (0–1 minimum to maximum) [23]. The short form International Physical Activity Questionnaire (IPAQ) was used to assess physical activity levels [24]. Physical activity was categorised according as follows: (a) high: vigorous exercise at least 20 min/day, three days/week, (b) moderate: moderate intensity activity 30 min/day, five days/week or (c) low: five or more days of any combination of walking, moderate/vigorous intensity activities of a minimum of 600 MET-min/week [25].

#### Work-related measures

These included occupation category, industry type, hours worked in the previous seven days, daily work-related computer use, and ergonomic workstation evaluation scores. Workplace psychosocial factors were assessed using the 18-item version of the Job Content Questionnaire (JCQ) [26, 27]. The total scores for each of the four domains (psychological job demands [three items], physical job demands [two items], job control [nine items], and social support from supervisors and colleagues [four items]) were used. Health beliefs were assessed with three questions from the Fear-Avoidance Beliefs Questionnaire (FABQ) [28]. Each item was scored on a seven-point scale from “completely disagree” (score of zero) to “completely agree” (score of seven). Job Satisfaction was evaluated with a single item using the Kunin faces measure [29, 30].

### Intervention adherence and maintenance

Adherence during the 12-week intervention period was recorded by the intervention physiotherapist as the proportion (%) of supervised exercise sessions (13 sessions) attended for the EET group, and the proportion (%) of health promotion sessions (12 sessions) recorded (by the session facilitator) for the EHP group. Adherence at the 12-month follow-up was recorded via an online self-report survey administered monthly and was categorised as either regular (at least once/week) or irregular/none (less frequently than once/week) practice of exercise training (EET group) or lifestyle changes (EHP) during the last four weeks.

### Adverse events

Adverse events were assessed weekly by the visiting intervention physiotherapist and reported to study staff in-between assessments. An adverse event was accepted as one that required reporting as per the institutional Occupational Health and Safety requirements – an incident, injury or near miss.

### Statistical analyses

An Intention-to-treat (ITT) analysis which includes all participants regardless of their level of participation was undertaken. Two separate statistical analyses were performed: 1) All Workers that included all participants, and 2) the subgroup of office workers identified as neck cases labelled ‘Neck Cases’ (symptomatic of neck pain ≥3 at baseline). To examine the intervention effects, only those participants with data at all three time points (baseline, 12 weeks and 12 months) were included to form the analytical sample. To examine the potential impact of this approach, differences between the analytical and full sample for each intervention arm were checked using 2-sample t-tests for continuous variables, and Chi-square statistics for categorical variables, and reported as mean ± SD or observed proportions (%) respectively.

Differences between the two intervention groups were compared using Pearson’s Chi-square test for categorical variables or ANOVA tests for continuous variables. Potentially confounding variables (demographic, general and global health, work-related, physical) observed to have a potential univariate relationship with a change in neck pain intensity (pre-post intervention), indicated by *p* < 0.1, were tested in the multivariate model, as were interactions. Neck pain intensity over the three time points (baseline, 12 weeks, 12 months) was analysed using the Generalized Estimating Equations (GEE) approach separately for the general office worker and neck cases subgroup samples. An autogressive working covariance matrix was used to account for correlation and dependence between repeated measurements on the same individual over time while accounting for any potential confounding variables identified as having a univariate relationship with the outcome (change in neck pain). A *p*-value of < 0.05 was accepted as statistically significant. Stata statistical program (version 15, StataCorp, College Station, TX, USA) was used to conduct the analyses.

## Results

### Flow of participants through the study

**Fig. 1:**
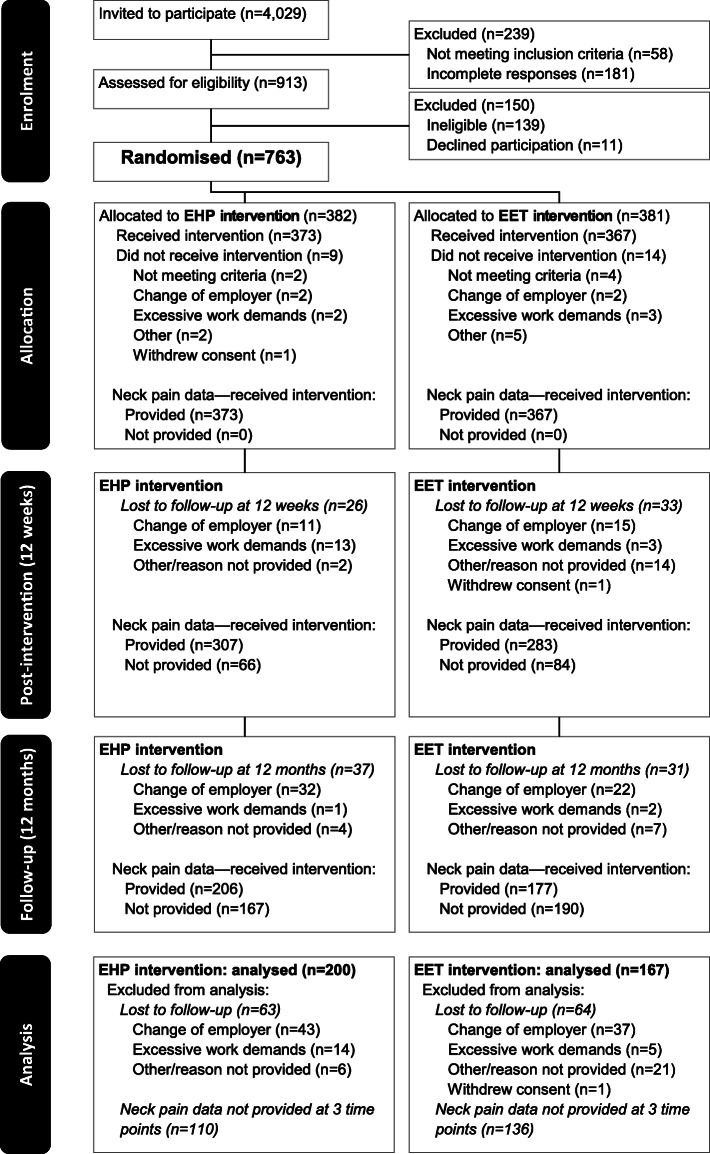
Study flow of trial participants. The number of participants reported at each follow-up reflects those completing assessments for the secondary outcome of interest (i.e., neck pain intensity) at all 3 time points

The participant flow through the study is presented in Fig. 1. Of the 4029 employees approached, 23% volunteered and were assessed for eligibility with 84% randomized to an intervention arm in 100 clusters. Of the 763 employees recruited and allocated to an intervention arm, 23 discontinued their participation prior to the commencement of intervention sessions, leaving 740 participants who commenced their allocated intervention.

Of these 740 participants (Supplementary Table A), 367 participants (49.59%) returned requisite data at three time points including those in EET (*n* = 167) and EHP (*n* = 200) resulting in 1101 pain observation points. Baseline characteristics of the participants included in the general office worker (EET *n* = 167, EHP n = 200) and neck case (EET *n* = 41, EHP *n* = 55) analyses are listed in Table 1 grouped according to intervention. This reduced sample was found on the whole to be statistically similar to the excluded participants (Supplementary Table B) with the only significant differences in baseline characteristics only being lower total hours worked in the previous seven days and psychological distress while mean age was slightly greater in the analysed sample.

**Table 1:** Baseline characteristics of participants by intervention group for All Workers and Neck Cases

Variable	All Workers (***N*** = 367)		Neck Cases (***N*** = 96)	
	EHP (***N*** = 200)	EET (***N*** = 167)	EHP (***N*** = 55)	EET (***N*** = 41)
**Demographic**				
Age, years mean ± SD	44.1 ± 10.2	42.9 ± 10.4	43.6 ± 9.5	45.5 ± 10.9
Gender, females n (%)	123 (61.5)	105 (62.9)	40 (73.0)	34 (83.0)
Body mass index, kg/m^2^ mean ± SD	27.3 ± 5.5	26.9 ± 5.3	26.3 ± 4.1	25.5 ± 4.7
Highest Level of Education n (%)				
Primary to Year 12	46 (23.0)	39 (23.4)	10 (18.0)	8 (20.0)
University	131 (65.5)	99 (59.3)	37 (67.0)	23 (56.0)
Trade College	23 (11.5)	29 (17.4)	8 (5.0)	10 (24.0)
**Workplace Measures**				
Occupational category n (%)				
Manager/senior official	40 (20.0)	23 (13.8)	12 (22.0)	5 (12.0)
Professional, associate professional, technical or other	93 (46.5)	84 (50.3)	25 (45.0)	21 (51.0)
Administrative, secretarial, or personal services	67 (33.5)	60 (35.9)	18 (33.0)	15 (37.0)
Industry n (%)				
Local Government	16 (8.0)	11 (6.6)	3 (5.0)	4 (10.0)
State Government	81 (40.5)	64 (38.3)	25 (45.0)	15 (37.0)
Federal Government	33 (16.5)	22 (13.2)	10 (18.0)	6 (15.0)
Private	47 (23.5)	50 (29.9)	11 (20.0)	12 (29.0)
Other	23 (11.5)	20 (12.0)	6 (11.0)	4 (10.0)
Total hours worked in previous 7 days mean ± SD	38.3 ± 10.2	38.3 ± 8.2	39.1 ± 9.7	37.6 ± 6.9
Time using computer at work n (%)				
< 6 h/day	32 (16.0)	30 (18.0)	8 (15.0)	10 (24.0)
≥ 6 h/day	168 (84.0)	137 (82.0)	47 (85.0)	31 (76.0)
Total ergonomic score mean ± SD	**30.5 ± 2.9**	**31.4 ± 2.7**	**30.0 ± 2.9**	**31.2 ± 3.0**
**Health Behaviour Measures**				
Received workers’ compensation for neck/shoulder symptoms in previous 12 months n (%)	0 (0.0)	1 (0.6)	0 (0)	0 (0)
Sought healthcare professional for neck/shoulder symptoms in previous 12 months n (%)	66 (33.0)	51 (30.5)	29 (53.0)	24 (59.0)
Taken medication for neck/shoulder symptoms in previous 4 weeks n (%)	30 (15.0)	28 (16.8)	18 (33.0)	16 (39.0)
Total number of comorbidities mean ± SD	**0.8 ± 1.0**	**0.6 ± 0.9**	**0.9 ± 1.0**	**0.5 ± 0.8**
**Psychosocial Measures**				
Job Content Questionnaire mean ± SD				
Psychological Job Demands	8.5 ± 2.5	8.3 ± 2.5	9.0 ± 2.9	8.5 ± 2.5
Physical Job Demands	3.2 ± 1.2	3.4 ± 1.3	3.2 ± 1.5	3.6 ± 1.3
Job Control	21.5 ± 3.5	21.1 ± 2.8	21.3 ± 3.7	20.7 ± 3.1
Social Support	15.6 ± 2.2	15.8 ± 2.5	15.6 ± 2.2	15.2 ± 3.1
Psychological distress mean ± SD	3.6 ± 2.9	3.1 ± 2.8	4.4 ± 3.3	3.2 ± 2.8
Health beliefs mean ± SD	3.7 **±** 1.5	3.5 **±** 1.6	**3.9 ± 1.4**	**3.3 ± 1.7**
Job satisfaction mean ± SD	4.9 **±** 1.1	5.0 ± 1.0	4.8 ± 1.0	4.8 ± 1.2
Health-related Quality of Life mean ± SD	0.8 ± 0.1	0.8 ± 0.1	**0.8 ± 0.1**	**0.8 ± 0.1**
IPAQ n (%)				
Low	74 (37.0)	60 (35.9)	20 (36.4)	14 (34.2)
Moderate	109 (54.5)	88 (52.7)	32 (58.2)	23 (56.1)
High	17 (8.5)	19 (11.4)	3 (5.5)	4 (9.8)
**Intervention Adherence**				
12 weeks - % Supervised sessions attended mean ± SD	67.4 ± 20.7	70.9 ± 21.1	69.7 ± 15.8	70.0 ± 19.1
12 months – Participants regularly exercising (EET) or practicing lifestyle changes (EHP) n (%)	125 (62.5)	25 (15.4)	38 (69.1)	8 (21.1)

boldface = significant between-group (i.e. EET & EHP) differences at *p*-value < 0.05

*EET*  ergonomic and exercise intervention group, *EHP*   ergonomic and health promotion intervention group, *SD*  standard deviation

Table 1 shows that at baseline, there were only a few significant between-group differences of minor magnitude, including the total workstation ergonomic score and number of comorbidities in All Workers and Neck Cases. Only for Neck Cases though, there were significant differences at baseline in health beliefs and health-related quality of life.

### Intervention adherence

During the intervention period, observed adherence (% of attended sessions) was similar for the intervention groups in both All Workers and Neck Cases (Table 1). At the end of the maintenance period (12-month follow up), the proportion of participants reporting regular (at least once/week) exercise (EET group) or lifestyle change (EHP) participation was particularly low for the EET group in both groups.

### Intervention effects

**Fig. 2:**
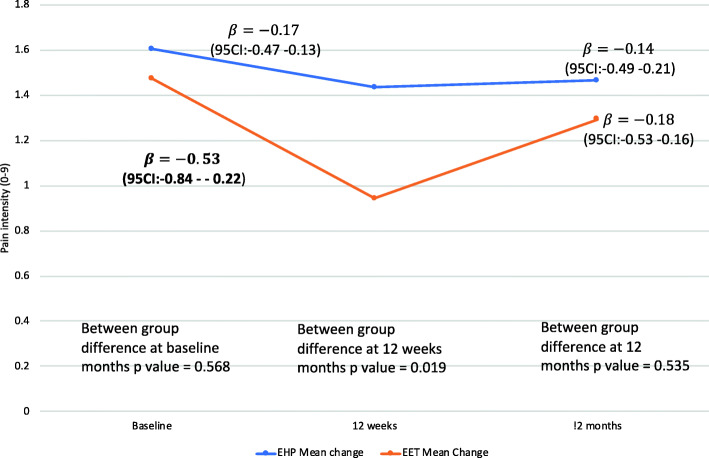
Adjusted neck pain intensity (mean) between- and within-groups over time for All Workers (ITT analysis *n* = 367). Model adjusted for those variables significant in the univariate association (gender; age; comorbidity; interaction between age and comorbidity; medication use and seen by a professional; and total hours worked)

**Fig. 3:**
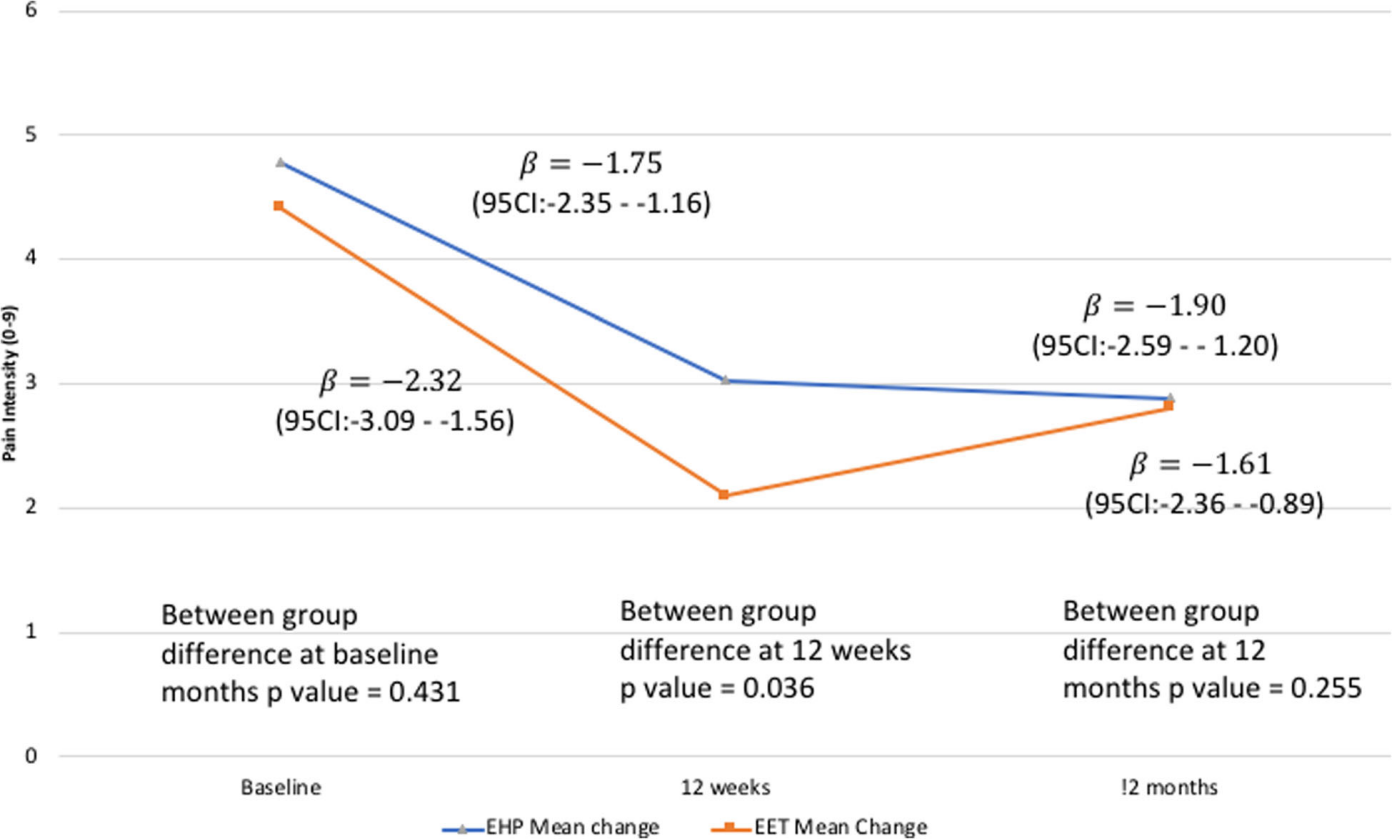
Adjusted neck pain intensity (mean) between- and within-groups over time for Neck Cases (ITT analysis *n* = 96). Model adjusted for those variables significant in the univariate association (gender; age; comorbidity; ergonomic score; interaction between age and comorbidity; medication use and seen by a professional interaction; total hours worked and group interaction)

The mean (±SD) of neck pain severity scores (adjusted) and percentage change from baseline at each time point for All Workers and Neck Cases is shown in Table 2. The adjusted models are presented for All Workers (Fig. 2) and Neck Cases (Fig. 3). Potential confounding variables shown to have a significant univariate relationship (*p* < 0.1) with a change in pain intensity (demographic, general and global health, work-related, variables) are listed below (no physical variables were retained in the final model) specific to the analyses for All Workers and Neck Cases. Findings for the unadjusted models are in the Supplementary Figs. 2A and 3A.

**Table 2:** Mean (± SD) of neck pain severity scores and adjusted percent change from baseline at each time point for the All Workers and Neck Cases

**All Workers** ***n*** **= 367**		
**Time point**	**EHP (*N*** **= 200)**	**EET (*N* = 167)**
**Baseline**	1.61 ± 2.21	1.47 ± 1.96
**12 weeks**	1.47 ± 2.15 8.7% reduction from baseline	1.02 ± 1.62 30.6% reduction from baseline
**12 months**	1.56 ± 2.16 3.1% reduction from baseline	1.35 ± 2.15 8.2% reduction from baseline
**Neck Cases (*n* = 96)**		
	**EHP (*N* = 55)**	**EET (*N* = 41)**
**Baseline**	4.78 ± 1.58	4.41 ± 1.53
**12 weeks**	2.96 ± 2.59 38.1% reduction from baseline	2.02 ± 2.01 54.2% reduction from baseline
**12 months**	2.76 ± 2.40 42.3% reduction from baseline	2.78 ± 2.57 37.0% reduction from baseline

#### All workers

Model findings were corrected to account for univariate associations including; gender; age; comorbidity; total hours worked and the significant interactions (age and comorbidity); (medication use and seen by a professional). Significant between-group differences in the reduction of neck pain intensity favouring the EET group were observed at 12 weeks (EET: β = − 0.53 points 95% CI: − 0.84– − 0.22) and EHP: β = − 0.17 points 95% CI: − 0.47–0.13), *p* = 0.019). Reductions in pain intensity were not maintained at 12 months with no between-group differences observed in the All Workers group (EET: β = − 0.18, 95% CI: − 0.53–0.16 and EHP: β = − 0.14 points 95% CI: − 0.49–0.21, *p* = 0.535) (Fig. 2).

#### Neck cases

Model findings were corrected to account for univariate associations including: gender; age; comorbidity; ergonomic score; and several interactions (age and comorbidity); (medication use and seen by a professional); (total hours worked and group). The EET intervention group demonstrated significantly greater reductions in neck pain at 12 weeks compared to the EHP group ((EET: β = − 2.32 points 95% CI: − 3.09– − 1.56 and EHP: β = − 1.75 points 95% CI: − 2.35– − 1.16, *p* = 0.036). Intervention groups were not statistically different at the 12-month follow-up; however, significant reductions in neck pain intensity for both groups were still evident from baseline (EET: β = − 1.61 points 95% CI: − 2.36– − 0.89 and EHP: β = − 1.9 points 95% CI: − 2.59– − 1.20, *p* = 0.255) (Fig. 3).

### Adverse events

Two participants reported musculoskeletal symptoms secondary to physical capacity testing (*n* = 1) and exercise training (*n* = 1). These participants were followed up by a physiotherapist and completed their interventions without further issue.

## Discussion

The findings partly support our hypothesis that a combined ergonomics and exercise training intervention may be more effective than a combined ergonomics and health promotion intervention in reducing neck pain intensity in All Workers as well as the subgroup of Neck Cases. The EET group had experienced significantly greater reductions in neck pain compared to the EHP group by the end of the intervention period (12 weeks) in both the All Workers and Neck Cases confirming the findings of previous systematic reviews [3, 4]. However these between-group differences were absent at 12-month follow-up. As evident in Figs. 2 and 3, this loss of between-group differences at 12 months reflects a progressive return of pain intensity in the EET group. Interestingly, this is matched by very poor adherence at 12 months with < 21% of the EET group (Table 1) reporting the continuation of regular exercise. It is possible that higher participation rates would have yielded greater reductions in neck pain with evidence of a dose-response relationship with participation in exercise training for office workers [3]. The findings in a previous study evaluating resistance exercise of the neck/shoulder region demonstrated sustained improvements in neck/shoulder pain when exercise training was maintained over an intervention period of 12 months [31]. Such findings support the notion that the benefits of exercise may be greatest when exercise is continued in the long-term [32], particularly given the recurrent nature of neck pain and its disability burden to society [33, 34]. Previous studies support the importance of management support and organisational commitment to ensure the success of such interventions [35]. However, in a process evaluation of this trial, strong organisational commitment did not translate to implementation effectiveness [36]. Nevertheless, this study showed that strength training could be successfully implemented during working hours but that strategies are needed to support adherence in the long-term.

Of relevance to industry, reductions in neck pain intensity in the subgroup of Neck Cases were clinically meaningful after 12 weeks (EET 2.39, 54%; EHP 1.82, 38%, both > 30% reduction) [19]. These reductions were of greater magnitude to those reported in previous studies (0.7–1.6 point pain reduction on a 0–9 scale [31]) using similar interventions and participants with similar (or lower) levels of neck pain at baseline (3.3–4.8 points) [31, 37]. Reduction in neck pain intensity in All Workers in both intervention groups in our study (0.14–0.45, [8.7–30.6%] at 12 weeks; 0.05–0.12 [3–8%] at 12 months) was also similar to previous studies including all office workers (0.4 and 0.6 points reduction) [37, 38]. However, the advantage of our intervention is that these benefits were gained after a 12 week rather than 20-week [37] or a one-year intervention of similar exercises [31]. Notably, it was challenging to achieve large amplitude reductions in neck pain intensity in All Workers who reported low mean pain intensity at baseline (EET 1.47, EHP 1.61) reflecting the dilution effect of combining workers with and without neck pain at baseline [38]. Importantly, the magnitude of effect on neck pain intensity observed in exercise intervention participants at 12 months in the present and previous studies involving All Workers is similar to that achieved by our EHP intervention group a potentially more scalable and financially efficient alternative for employers wishing to provide a service suitable for all workers. However, the potential gains in health must be considered with the evidence that the EET intervention delivered productivity benefits for All Workers in the long-term compared to the EHP [14].

The ergonomic component included modifications to items within the workstation and postural advice. It is possible that this component may have contributed to the significant, but similar improvements in neck pain experienced by both intervention groups at 12-month follow-up. Similar results were reported for the combination of ergonomic modification and stretching exercise [9] and ergonomic training and neck motor control training [39] in symptomatic office workers. The lack of a third intervention group comprising only the workstation ergonomic component limited the ability to exclude potential individual or work-related reasons associated with changes in pain in both groups. However, an ergonomic intervention alone has not been shown to be as effective as the combination with exercise [9]. It is possible that more regular follow-up was needed to achieve further improvements or the addition of ergonomic educational training. Pillastrini et al. [40] demonstrated that a personalized comprehensive ergonomic intervention with twice monthly follow-up for five months plus training reduced the proportion of office workers with neck pain compared with an ergonomic brochure alone. However, such an intensive intervention may not be financially viable in the long-term.

While we hypothesized that the EET training would be more effective than the EHP, it is possible that some features of the latter intervention contributed to similar reductions in neck pain at 12 months. For example, the health promotion sessions were designed to vary week-to-week and be engaging and social, while EET participants received the same exercise protocol each week, with only the exercise intensity varying on a weekly basis. The health promotion sessions could have motivated individuals to improve lifestyle behaviours and mental health, thus contributing to improvements in self-reported pain. It is also possible that an hour at a time away from work provided additional benefit to mental and physical stressors, which have been associated with neck symptoms [41]. Potentially, the inclusion of the health promotion intervention offers advantages to industry as a realistic alternative to exercise. Similar studies testing exercise interventions have included options of no intervention or general health counselling risking poor adherence. The adherence to the health promotion intervention in our study was 62% at 12 months for All Workers while it was only 9% for those receiving health counselling in a similar study [31]. Further exploration of the potential mechanisms underlying the observed improvements in neck pain and our previously reported productivity benefits [14] across both groups is warranted.

### Study strengths and limitations

Important strengths of this study were the clustered design using ITT analyses, and 12-month follow-up. The sample size permitted separate analyses of All Workers and Neck Cases, allowing generalizability of results to both office worker populations. Furthermore, this study tested two multi-component interventions, which are considered more likely to demonstrate benefit than single-component interventions.

Limitations include the low baseline severity of neck pain in the All Workers group, but providing access to health interventions to all employees is important from a primary prevention perspective. Interpretation of results is restricted by the missing data due to low compliance at follow-up. Whilst not desirable, the 12-month data loss is not unusual compared to other lifestyle workplace-based trials (approximately 45–65%) [37, 42–45]. In this trial, several attempts were made to contact participants who did not provide data at 12-week and 12-month follow-up. The trial’s conservative definition of neck cases (pain intensity ≥3 last seven days) and the recruitment of participants who were unaware of the trial intention may have resulted in the recruitment of lower than expected numbers of neck cases. Organisations were asked not to undertake other workplace-based interventions during the study period, but this was only assessed retrospectively.

## Conclusions

A combined exercise and ergonomic intervention was more effective than a combined health promotion and ergonomic intervention in reducing neck pain intensity in All Workers and those with neck pain immediately following the intervention period. However, intervention differences were not maintained at 12-month follow-up suggesting the potential need for exercise interventions to be long-term or continuous to maintain benefits. This study provides evidence for the benefit for the health promotion intervention to be a scalable and financially efficient program to address neck pain in all office workers although productivity gains may be greater for those participating in exercise interventions. Strategies to promote adherence to exercise in the long-term may deliver both health and productivity benefits.

## Supplementary Information

**Additional file 1.**

## Data Availability

The datasets used and/or analysed during the current study are available from the corresponding author on reasonable request.

## Abbreviations

EET: ergonomic and exercise training
EHP: ergonomic and health promotion
