# Supplementary material for: A cluster-randomized trial of workplace ergonomics and neck-specific exercise versus ergonomics and health promotion for office workers to manage neck pain – a secondary outcome analysis
Source: BMC Musculoskelet Disord. 2021 Jan 12;22:68. doi: 10.1186/s12891-021-03945-y (PMC7805092; doi:10.1186/s12891-021-03945-y)
Supplement: Supplementary file 1 — Additional file 1. [file 12891_2021_3945_MOESM1_ESM.docx]

**Supplementary Material**

Table A. Baseline characteristics of all participants by intervention group for All Workers and for the subgroup of Neck Cases.

| **Variable** | **All Workers**  **(N=740)^*^** | | **Neck Cases**  **(N=200)^*^** | |
| --- | --- | --- | --- | --- |
|  | **EHP (N=373)** | **EET (N=367)** | **EHP (N=109)** | **EET (N=91)** |
| Age, years | 43.0 ± 10.3 | 42.4 ± 11.1 | 42.3 ± 9.5 | 43.7 ± 11.6 |
| Gender (female) | 230 (61.7) | 221 (60.2) | 76 (69.7) | 72 (79.1) |
| Body mass index (kg/m^2^) | 27.0 ± 5.4 | 27.1 ± 6.1 | 26.3 ± 4.8 | 26.4 ± 5.8 |
| Occupational category |  |  |  |  |
| Manager/senior official | 75 (20.1) | 67 (18.3) | 21 (19.3) | 15 (16.5) |
| Professional, associate professional, technical or other | 173 (46.4) | 173 (47.1) | 54 (49.5) | 42 (46.2) |
| Administrative, secretarial, or personal services | 125 (33.5) | 127 (34.6) | 34 (31.2) | 34 (37.4) |
| Industry |  |  |  |  |
| Local Government | 26 (7.0) | 28 (7.6) | 8 (7.3) | 8 (8.8) |
| State Government | 152 (40.8) | 157 (42.8) | 47 (43.1) | 39 (42.9) |
| Federal Government | 52 (13.9) | 49 (13.4) | 16 (14.7) | 10 (11.0) |
| Private | 100 (26.8) | 97 (26.4) | 28 (25.7) | 26 (28.6) |
| Other | 43 (11.5) | 36 (9.8) | 10 (9.2) | 8 (8.8) |
| Highest Level of Education |  |  |  |  |
| Primary to Year 12 | 80 (21.5) | 87 (23.7) | 20 (18.4) | 17 (18.7) |
| University | 238 (63.8) | 218 (59.4) | 74 (67.9) | 55 (60.4) |
| Trade College | 55 (14.8) | 62 (16.9) | 15 (13.8) | 19 (20.9) |
| Received workers’ compensation for neck/shoulder symptoms in previous 12 months | 2 (0.5) | 2 (0.5) | 2 (1.8) | 0 (0) |
| Taken medication for neck/shoulder symptoms in previous 4 weeks | 63 (16.9) | 62 (16.9) | 40 (36.7) | 33 (36.3) |
| Sought healthcare professional for neck/shoulder symptoms in previous 12 months | 122 (32.7) | 106 (28.9) | 57 (52.3) | 50 (55.0) |
| Total hours worked in previous 7 days | 39.6 ± 9.6 | 39.7 ± 9.3 | 40.0 ± 9.2 | 40.4 ± 8.8 |
| Time using computer at work |  |  |  |  |
| <6 hours/day | 57 (15.3) | 67 (18.3) | 15 (13.8) | 16 (17.6) |
| ≥6 hours/day | 316 (84.7) | 300 (81.7) | 94 (86.2) | 75 (82.4) |
| Total ergonomic score | **30.8 ± 3.0** | **31.3 ± 2.8** | **30.3 ±3.2** | **31.2 ± 2.6** |
| Total number of comorbidities | **0.7 ± 1.0** | **0.5 ± 0.8** | 0.8 ± 1.0 | 0.6 ± 0.9 |

^*^ Data are number and percentage of participants n (%) or mean ± SD
boldface= significant between-group (i.e. EET & EHP) differences at p-value < 0.05
EET= ergonomic and exercise intervention group
EHP= ergonomic and health promotion intervention group
SD= standard deviation

Table B: Comparison of the analysed sample with those excluded at Baseline 3 months and 12 months.

| **Variable** |  | |  |
| --- | --- | --- | --- |
|  | **Excluded (N=373)** | **Analysis Sample***  **EHP (N=367)** | **p-value** |
| **Demographic** |  |  |  |
| Age, years | 41.8 ± 11.0 | 43.6 ± 10.3 | **0.050** |
| Gender (female) | 223 (59.8) | 228 (2.1) | 0.514 |
| Body mass index (kg/m^2^) | 27.0 ± 6.0 | 27.1 ± 5.5 | 0.697 |
| Highest Level of Education |  |  |  |
| Primary to Year 12 | 82 (22.0) | 85 (23.2) | 0.476 |
| University | 226 (60.6) | 230 (62.7) |  |
| Trade College | 65 (17.4) | 52 (14.2) |  |
|  |  |  |  |
| **Workplace Measures** |  |  |  |
| Occupational category |  |  |  |
| Manager/senior official | 79 (21.2) | 63 (17.2) | 0.376 |
| Professional, associate professional, technical or other | 169 (45.3) | 177 (48.2) |  |
| Administrative, secretarial, or personal services | 125 (33.5) | 127 (34.6) |  |
| Industry |  |  |  |
| Local Government | 27 (7.2) | 27 (7.4) | 0.629 |
| State Government | 164 (44.0) | 145 (39.5) |  |
| Federal Government | 46 (12.3) | 55 (15.0) |  |
| Private | 100 (26.8) | 97 (26.4) |  |
| Other | 36 (9.65) | 43 (11.7) |  |
| Total hours worked in previous 7 days | 41.0 ± 9.4 | 38.3± 9.3 | **<0.001** |
| Time using computer at work |  |  |  |
| <6 hours/day | 62 (16.6) | 62 (16.9) | 0.921 |
| ≥6 hours/day | 311 (83.4) | 305 (83.1) |  |
| Total ergonomic score | 31.1 ± 2.9 | 31.0 ± 2.8 | 0.441 |
|  |  |  |  |
| **Health Behaviour Measures** |  |  |  |
| Received workers’ compensation for neck/shoulder symptoms in previous 12 months | 3 (0.8) | 1 (0.3) | 0.324 |
| Taken medication for neck/shoulder symptoms in previous 4 weeks | 67 (18.0) | 58 (15.8) | 0.433 |
| Sought healthcare professional for neck/shoulder symptoms in previous 12 months | 111 (29.8) | 117 (31.9) | 0.532 |
| Total number of comorbidities | 0.6 ± 0.9 | 0.7 ± 1.0 | 0.114 |
|  |  |  |  |
| **Psychosocial Measures** |  |  |  |
| Job Content Questionnaire |  |  |  |
| Psychological Job Demands | 8.9±2.6 | 8.4±2.5 | 0.075 |
| Physical Job Demands | 3.3±1.3 | 3.3 ±1.3 | 0.710 |
| Job Control | 21.5 **±** 3.6 | 21.3±3.2 | 0.400 |
| Social Support | 15.3±2.7 | 15.7±2.4 | 0.055 |
| Psychological Distress | **4.0** ± 3.6 | 3.4 ± 2.9 | **0.004** |
| Health beliefs | 3.7 **±**1.6 | 3.6 **±**1.5 | 0.123 |
| Job Satisfaction | 4.9 ± 1.1 | 4.9 **±**1.1 | 0.367 |
| Health Related Quality of Life | 0.8 ±0.1 | 0.8 ±0.1 | 0.642 |
| IPAQ |  |  |  |
| Low | 120(32.2) | 134 (36.5) | 0.391 |
| Moderate | 209(51.5) | 197 (53.7) |  |
| High | 44 (11.8) | 36 (9.8) |  |

*Analysis sample includes those with data at 3 time points

Data are number and percentage of participants n (%) or mean ± SD
EET= ergonomic and exercise intervention group
EHP= ergonomic and health promotion intervention group


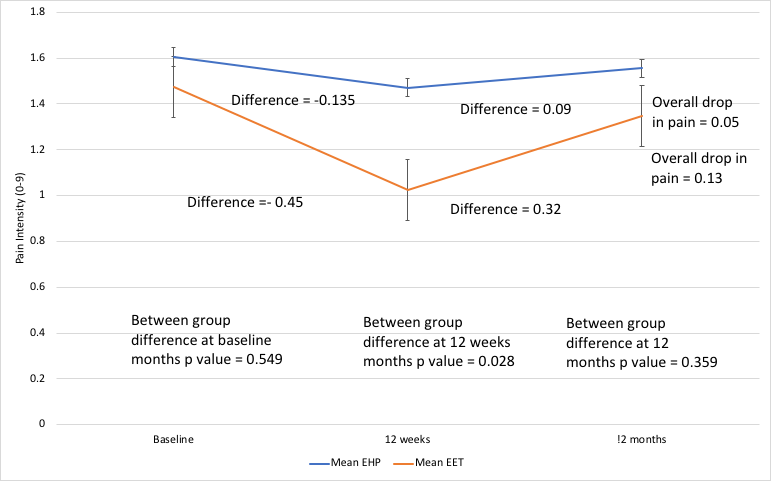


**Figure 2A.** Neck pain intensity (unadjusted) (mean ± SD) between- and within-groups over time for All Workers (ITT analysis; n=367)

Note: The ‘Difference’ represents mean change in pain between time points


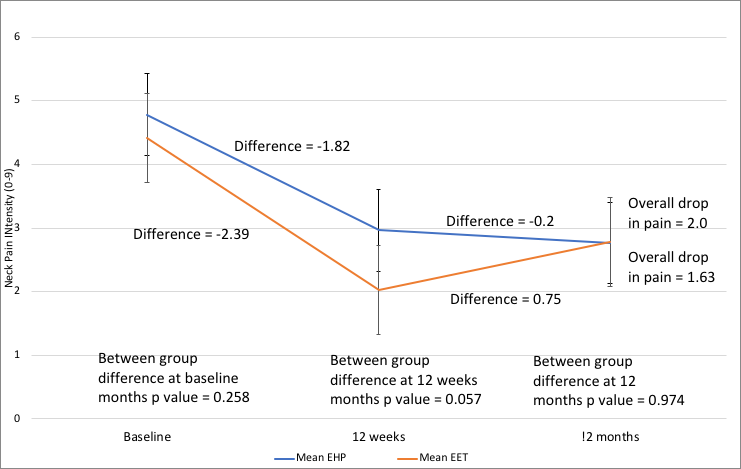


Figure 3A. Neck pain intensity (unadjusted) (mean ± SD) between- and within-groups over time for the Neck Cases (ITT analysis n=96).

Note: The ‘Difference’ represents mean change in pain between time points
